# Benefits, Problems, and Potential Improvements in a Nationwide Patient Portal: Cross-sectional Survey of Pharmacy Customers’ Experiences

**DOI:** 10.2196/31483

**Published:** 2021-11-03

**Authors:** Maria Sääskilahti, Anna Ojanen, Riitta Ahonen, Johanna Timonen

**Affiliations:** 1 School of Pharmacy Faculty of Health Sciences University of Eastern Finland Kuopio Finland; 2 Humalisto Pharmacy Turku Finland

**Keywords:** benefit, problem, improvement need, patient portal, patient engagement, experience, survey

## Abstract

**Background:**

Patient engagement is a worldwide trend in health care. Patient portals have the potential to increase patients’ knowledge about their health and care and therefore enhance patient engagement. Portal users’ experiences are needed to determine if these portals work appropriately and if there are barriers to achieving the aims that were set before their implementation.

**Objective:**

The aim of this study is to analyze pharmacy customers’ experiences of the Finnish nationwide patient portal My Kanta in terms of benefits, problems, and potential improvements.

**Methods:**

A questionnaire survey was conducted among pharmacy customers in the spring of 2019. The questionnaires (N=2866) were distributed from 18 community pharmacies across mainland Finland to customers aged ≥18 years who were purchasing prescription medicines for themselves or their children aged <18 years. Using open-ended questions, customers were asked about their experiences of the benefits and problems of My Kanta and what improvements could be made. Their responses were encoded and categorized using inductive content analysis, stored in SPSS Statistics for Windows, and analyzed using frequencies.

**Results:**

Of the 2866 questionnaires, a total of 994 (34.68%) questionnaires were included in the analysis. Most respondents were My Kanta users (820/994, 82.5%); of these 820 users, 667 (81.3%) reported at least one benefit, 311 (37.9%) reported at least one problem, and 327 (39.9%) reported at least one potential improvement when using My Kanta. The most commonly mentioned benefits were opportunities to view health data (290/667, 43.5%) and prescriptions (247/667, 37%) and to renew prescriptions (220/667, 33%). The most extensively reported problems with My Kanta were that the portal lacks health data (71/311, 22.8%), navigating the service and searching for information is difficult (68/311, 21.9%), and the delay before health data are incorporated into the service (41/311, 13.2%). The most frequently suggested potential improvements were that My Kanta needs more comprehensive health data (89/327, 27.2%); the service should be easier to navigate and information easier to access (71/327, 21.7%); the service should have more functions (51/327, 15.6%); and health data should be entered into the portal more promptly (47/327, 14.4%).

**Conclusions:**

Pharmacy customers reported more benefits than problems or potential improvements regarding the use of My Kanta. The service is useful for viewing health data and prescriptions and for renewing prescriptions. However, portal users would like to see more data and functions available in the portal and data searches to be made easier. These improvements could make the data and functions provided by the portal easier to view and use and hence promote patient engagement.

## Introduction

### Background

Promoting patient engagement is a worldwide trend, with the goal of improving patient-centered care and providing greater safety in health care [1]. The opportunity to access one’s own medical data is regarded as a key element in patient engagement, as it provides patients with more information about their own health and care. In recent decades, many countries have developed national eHealth strategies, including the implementation of electronic health record systems [2]. Some of these systems include an opportunity for patients to view their electronic health records via patient portals. However, enhancing patient engagement requires not only implementing patient portals for data viewing but also ensuring that these services are user-friendly and can be used appropriately [1]. Collecting data regarding patients’ perceptions, needs, and experiences when updating patient-centered health services is also part of patient engagement. In the case of patient portals, it is important to understand how patients use the medical data provided and whether the portal has achieved the goals set before its implementation. The aims of portals are to increase patients’ empowerment and thus encourage them to take more responsibility for their own health and care.

As patient portals have only been introduced in recent years, only a limited number of studies have investigated users’ experiences of their usability. However, some studies have been conducted, mainly in the United States and the Netherlands, where patient portals are targeted at certain regions, diseases, or organizations [3-7]. On the other hand, few studies have investigated nationwide patient portals [8-10], partly because they have been implemented in only a few countries, mainly Nordic countries. Studies focused on users’ experiences of patient portals have shown that users are mostly satisfied with these portals [6-10]. Commonly reported benefits that portals have provided for their users are that medical records are viewable and can be read before or after visits and that users find themselves better informed and more empowered and engaged in their own care [3-7,9-11]. Being able to communicate with health care professionals has also been viewed as beneficial [3-6,11]. The problems encountered when using portals are mostly related to difficulty in understanding the information recorded within the portal and to the log-in process [3-5,10,11]. Users have reported that the portals could be improved by including more information, facilitating faster entry of data into the portals, and making the information more understandable [4,8-11]. Previous studies seem to indicate that experiences of patient portal use are fairly similar regardless of whether the portal is nationwide or specific to a certain area, disease, or organization.

### Study Context

Kanta Services is a Finnish nationwide entity providing services such as Patient Data Repository and Prescription Center into which health records and prescriptions recorded in all public and private health care units and pharmacies across Finland are saved [12]. Users of the services are health care professionals, pharmacists, and the public.

My Kanta, a nationwide patient portal, is part of Kanta Services and allows patients to access their health and prescription information [13]. Access to My Kanta requires a Finnish social security number and electronic authentication, such as a web banking code, an electronic ID, or a mobile certificate. My Kanta was initially introduced in 2010, and its content has been extended over the years [12,13]. In its early years, it only provided an opportunity to view e-prescriptions. Today, however, patients can browse their own or their dependents’ e-prescriptions and health data recorded as part of both public and private health care (Textboxes 1 and 2) [13,14,15]. Patients can also request prescription renewal and save their living will and organ donation testament. My Kanta also provides an opportunity to view in which health care units or pharmacies the user’s own data have been browsed or processed. We have reported a more detailed description of My Kanta and the Finnish e-prescription system in a previous study [16].

**Contents of My Kanta.**

**Contents**

- e-Prescriptions (all prescriptions have been issued electronically in Finland since 2017)
  Name, dosage, and indication of medicine
  Prescribing date and organization, prescriber
  Valid date of prescription
  Quantities of medicines outstanding
  Medicine purchases and dispensing events
  Prescription renewals
  Health care units and pharmacies where prescription information has been processed
- Health data
  Records of health care visits, diagnoses, critical risk factors
  Laboratory test results, x-ray examinations
  Referrals, medical certificates and reports
  Health care units where health records have been browsed

**Functions of My Kanta.**

**Functions**

- Viewing e-prescriptions and health data
- Printing out a summary of e-prescriptions
- Requesting a prescription renewal
- Giving informed consent to share health data
- Preventing the sharing of certain health data or e-prescriptions
- Saving a living will and organ donation testament
- Giving consent to other European pharmacies to dispense e-prescriptions (currently possible in Estonia, Croatia, and Portugal)
- Acting on behalf of dependents (at the time of the study, this refers to dependents aged <10 years. Access has only been extended gradually to underage dependents, aged 10-17 years, since October 2020).
  Viewing e-prescriptions and health data
  Submitting a prescription renewal request
  Giving informed consent to share a dependent’s health data

According to Kanta Services statistics, the My Kanta portal is widely used in Finland [17]. In 2019, more than 2 million people (total Finnish population 5.5 million) were using My Kanta, and the total number of log-ins was about 21 million. The familiarity of pharmacy customers with My Kanta, portal use, and experiences of its usability for viewing e-prescriptions were investigated in its early years in 2015 [18]. Since then, health data have been added to the portal, and portal use has increased significantly; hence, up-to-date studies are now needed. This study is part of a larger research project studying My Kanta use for viewing and monitoring health and prescription information from the patients’ perspective. We have previously reported on the use of different functions and the usability of the service surveyed by means of structured questions and have investigated the factors related to the use and nonuse of the service [16,19].

### Objectives

The aim of this study is to investigate pharmacy customers’ experiences of the benefits and problems of the Finnish nationwide patient portal My Kanta and how it could be improved.

## Methods

### Study Setting

A questionnaire survey was conducted in early spring 2019 of pharmacy customers aged ≥18 years who were purchasing prescription medicines for themselves or their children <18 years. This target group was chosen as we wanted to reach people who potentially have a need to use My Kanta. Questionnaires (Multimedia Appendix 1) were distributed in 18 community pharmacies from 6 regions across mainland Finland. Using convenience sampling, 1 university pharmacy branch (owned by a university but operating as a privately owned pharmacy), 1 large privately owned city pharmacy, and 1 small privately owned rural pharmacy were recruited from each region. The number of questionnaires delivered to each pharmacy was in proportion to the number of prescriptions dispensed annually by the pharmacy and ranged between 40 and 320. A total of 3560 questionnaires were mailed to the pharmacies. Pharmacists were instructed to inform all eligible customers about the survey after dispensing prescription medicines and to offer them the questionnaire. Pharmacies were not required to keep a record of customers who declined to participate. Customers filled in the questionnaires at home and mailed them in return envelopes to the research group. Questionnaires were handed out as long as there were forms left for a maximum of 2 weeks. After the study period, pharmacists reported the number of remaining questionnaires to allow the calculation of the response rate. Reminders could not be sent because the respondents’ personal data were not collected. Altogether, 2866 questionnaires were distributed.

### Questionnaire

The 4-page questionnaire included 22 structured, Likert-scale, and open-ended questions (Multimedia Appendix 1). The questionnaire consisted of 3 parts. The first part was for all respondents and concerned background information, the second part was for those respondents who used My Kanta, and the third part was for respondents who did not use My Kanta. The questionnaire was designed using My Kanta pages and previous surveys of patient portals [7,13,18,20-25]. It was initially tested for face validity by 3 researchers who were experienced in designing questionnaire surveys. Thereafter, the questionnaire and data collection procedure were piloted in a pharmacy, with minor revisions made as a result.

This study reports the results from 3 open-ended questions from the second part of the questionnaire. The questions were as follows: *What advantages or benefits has the use of My Kanta provided to you?* *What problems have you experienced when using My Kanta*? *How could My Kanta be improved to make it easier for you to monitor and manage your medication and health information?* We used open-ended questions to ask about the benefits, problems, and potential improvements to get an overview of issues that spontaneously come to respondents’ minds when they think about their experiences of My Kanta use. Background information was obtained by means of structured questions, except for the respondent’s year of birth and number of regularly used prescription medicines, which were obtained using open-ended questions.

### Data Analysis

A 2-phase analysis (qualitative and quantitative) was used. In the qualitative analysis, responses to open-ended questions were encoded and categorized using inductive content analysis. The analysis started by recording the answers in a table in Word 2016 (Microsoft Corp). This was continued to the point of saturation, which means that no new aspects related to the research questions emerged in the answers. After the saturation point, the remaining answers were examined, and only supplements to the previous aspects and new ideas were recorded. The saturation points for benefits, problems, and potential improvements were questionnaires 176, 248, and 250, respectively. In addition, if the answer to the question about benefits included a problem, it was moved to the analysis of problems, and if the answer to problems included an improvement idea, it was moved to the analysis of potential improvements. An analysis unit could be a single word, a sentence, or a group of sentences describing an idea related to benefits, problems, or potential improvements. Accordingly, an answer pertaining to more than 1 subject was separated into several analysis units. Simplifications were then made using these units. The simplifications were compared and then sorted into emerging subcategories, which were named according to all the simplifications in that subcategory. Similar subcategories were combined into main categories, and the main categories were named according to their content. Each questionnaire was then studied, and the responses were encoded into the main categories formed. The categorized data were stored in SPSS Statistics for Windows 10 (version 27.0; IBM Corp) for quantitative analysis. Data were analyzed using frequencies. Inductive content analysis was conducted by 2 researchers (AO and MS) after the simplifications were made. Contradictory categorizations were discussed by the research group. Content analysis was discussed by the research group throughout the process.

### Ethical Statement

Ethical approval required by the funding organization was granted by the Committee on Research Ethics of the University of Eastern Finland (statement 23/2018). Participation in the survey was voluntary. Filling in the questionnaire and mailing it to the research group were regarded as informed consent to participate. No incentives were provided to the participants. Pharmacy owners consented to handing out questionnaires in their pharmacies.

## Results

### Study Population

Altogether, 996 questionnaires were returned. Two questionnaires, however, were blank and were therefore excluded. Consequently, 34.68% (994/2866) of questionnaires were included in the study. Most respondents were female (687/994, 69.4%; Table 1). The mean age of the respondents was 62 (SD 14.484; range 18-99) years. Most respondents (820/994, 82.5%) were My Kanta users. The characteristics of My Kanta users were very similar to those of all respondents, with the exception of more frequent internet use and internet use to search for health-related information.

**Table 1:** Characteristics of the questionnaire respondents.

Characteristics			Total respondents (n=994), n (%)		My Kanta users (n=820), n (%)
**Gender^a^**			990 (99.6)		819 (99.9)
	Female	687 (69.4)		576 (70.3)	
	Male	303 (30.6)		243 (29.7)	
**Age (years)^a^**			958 (96.4)		791 (96.5)
	18-34	54 (5.6)		50 (6.3)	
	35-59	269 (28.1)		236 (29.8)	
	60-74	467 (48.7)		396 (50.1)	
	≥75	168 (17.5)		109 (13.8)	
**Education**					
	Basic education	185 (18.6)		129 (15.7)	
	Secondary education	523 (52.6)		444 (54.1)	
	University degree	286 (28.8)		247 (30.1)	
**Region^a^**			992 (99.8)		818 (99.8)
	Southern Finland	135 (13.6)		107 (13.1)	
	Southwestern Finland	144 (14.5)		109 (13.3)	
	Western and Central Finland	192 (19.4)		155 (18.9)	
	Eastern Finland	224 (22.6)		189 (23.1)	
	Northern Finland	222 (22.4)		193 (23.6)	
	Lapland	75 (7.6)		65 (7.9)	
**Internet use^a^**			987 (99.3)		814 (99.3)
	Daily or on several days a week	851 (86.2)		772 (94.8)	
	Once a week or less often	79 (8)		42 (5.2)	
	Not at all	57 (5.8)		0 (0)	
**Internet use to search for health-related information^a^**			991 (99.7)		819 (99.9)
	Yes	842 (85)		770 (94)	
	No	149 (15)		49 (6)	
**Chronic diseases diagnosed by a physician^a^**			982 (98.8)		809 (98.7)
	Yes	823 (83.8)		682 (84.3)	
	No	140 (14.3)		113 (14)	
	Does not know	19 (1.9)		14 (1.7)	
**Number of regularly used prescription medicines^a^**			942 (94.8)		780 (95.1)
	0	101 (10.7)		87 (11.2)	
	1-4	604 (64.1)		496 (63.6)	
	≥5	237 (25.2)		197 (25.3)	
**My Kanta use**					
	Yes	820 (82.5)		820 (100)	
	Has used it but is not going to use it anymore	21 (2.1)		0 (0)	
	Has never used it	153 (15.4)		0 (0)	

^a^Some of the respondents did not answer the question.

### Benefits

Of the My Kanta users, 81.3% (667/820) described at least one benefit that My Kanta use had provided (Table 2). The most frequently stated benefits were opportunities to view one’s own health data (290/667, 43.5%) and e-prescriptions (247/667, 37%) and to renew prescriptions (220/667, 33%). Many users (107/667, 16%) also thought it was useful in general that their own information was viewable via the service.

**Table 2:** Benefits that My Kanta has provided for its users.

Benefits^a^			Respondents (n=667), n (%)
**Health data viewable in the service**			290 (43.5)
	Viewing health data	—^b^	
	Viewing laboratory results	—	
	Viewing records of health care visits	—	
**e-Prescriptions viewable in the service^c^**			247 (37)
	Monitoring e-prescriptions	—	
	Monitoring validity of prescriptions	—	
	Monitoring amounts of outstanding medicines	—	
	Monitoring the need for prescription renewals	—	
	Prescriptions are in the service	—	
**Prescription renewals**			220 (33)
	Opportunity to renew prescriptions	—	
	Ease of prescription renewing	—	
**One’s own information viewable in the service**			107 (16)
	One’s own information can be viewed or checked	—	
	Ease of viewing one’s own information	—	
	One’s own information viewable anytime or anywhere	—	
	One’s own information viewable at home	—	
	All information in one place	—	
**Fever contacts and calls to health services**			42 (6.3)
	Fewer calls	—	
	No need to visit a physician	—	
	No need to visit a health care center or pharmacy	—	
**Easy to take care of health-related matters**			35 (5.2)
	Easy	—	
	Can be used anytime or anywhere	—	
	Can be used at home	—	
	Saving time	—	
**Other**			58 (8.7)
	Printing out information	—	
	Saving organ testament	—	
	Saving living will	—	
	Acting on behalf of others	—	

^a^From each main category, the most common subcategories are reported in the table.

^b^Not available.

^c^In Finland, all prescriptions are issued electronically.

The main category *Health data viewable in the service* included responses concerning laboratory results, records of health care visits, and health data in general (Table 2). It was thought useful that laboratory results, records of health care visits, and health data in general were viewable and could be easily monitored via the service. The respondents stated that by using My Kanta, they could keep up to date with their health information. They also found it useful, after their health care visit, to be able to check what had been discussed during the visit or what the physician had recorded.

The most frequent response in the main category *E-prescriptions viewable in the service* was that e-prescriptions could be (easily) viewed and monitored via the service (Table 2). Some users said that they kept a check on how long e-prescriptions were valid, whether there were any medicines outstanding, or whether e-prescriptions needed to be renewed. Some users found it useful that e-prescriptions were included in the service as there was no longer any need to keep paper versions and all prescriptions were safe in one place.

The main category *Prescription renewals* included responses that regarded the opportunity to renew prescriptions via the service as beneficial (Table 2). The renewal process was described as straightforward and fast.

In the main category *One’s own information viewable in the service*, the responses focused on the benefits of having access to one’s own information in general and that it was useful that information could easily be checked in one place, at anytime and anywhere (Table 2). Many respondents said that it was useful to be able to view this information in the privacy of their homes.

### Problems

More than 1 in every 3 users (311/820, 37.9%) described at least one problem in the use of My Kanta (Table 3). The most frequently mentioned problems were that the service lacked health data (71/311, 22.8%) and that users had difficulty navigating within the service and searching for information (68/311, 21.9%). Some users regarded the delay in health data being downloaded into the service to be a problem (41/311, 13.2%).

**Table 3:** Problems that users have experienced with My Kanta.

Problems^a^			Respondents (n=311), n (%)
**The service is lacking health data**			71 (22.8)
	Laboratory results not available	—^b^	
	Records of certain health care visits or units not available	—	
	Old data not available	—	
**Difficulty navigating the service and searching for information**			68 (21.9)
	Difficulty finding information	—	
	The service is badly organized	—	
	Problems switching between pages	—	
**Health data viewable with a delay**			41 (13.2)
	Health data downloaded into the service with a delay	—	
	Records of health care visits downloaded into the service with a delay	—	
	Laboratory test results downloaded into the service with a delay	—	
**Telecommunication problems**			33 (10.6)
	The service does not open properly	—	
	The service cuts off the user or is disrupted	—	
	Problems with the internet connection	—	
**User-driven challenges in using the service**			31 (10)
	Illnesses inhibit use of the service	—	
	Uncertainties about using computer or internet	—	
	Unaware of the content of the service	—	
	Difficulty understanding the health data recorded	—	
	My Kanta and other patient portals get mixed up	—	
**Inconveniences in logging in**			27 (8.7)
	Logging in is not possible without web banking codes	—	
	Web banking codes are not always carried	—	
	Web banking does not work properly	—	
	Logging in with web banking codes is laborious	—	
**Difficulty in prescription monitoring**			23 (7.4)
	Invalid and valid prescriptions become mixed	—	
	Difficulty understanding valid dates of prescriptions or amounts of outstanding medicines	—	
	Prescriptions cannot be arranged	—	
**Inconveniences in renewing prescriptions**			21 (6.8)
	Certain^c^ prescriptions cannot be renewed	—	
	Physician has not renewed the prescription	—	
	Problems choosing a health care unit for renewal	—	
**Incorrect information in the service**			13 (4.2)
	Information recorded incorrectly	—	
	Other persons’ data in the service	—	
	Difficulty correcting erroneous information	—	
**Other**			37 (11.9)
	Difficulty printing out e-prescriptions and health data	—	
	Guardian cannot see the data of dependents who are younger than 10 years	—	
	Uncertainties in data sharing procedures	—	

^a^From each main category, the most common subcategories reported in the table.

^b^Not available.

^c^For example, prescriptions issued in private health care or prescriptions issued over 28 months ago.

The main category *The service is lacking health data* included responses that My Kanta is lacking certain data (Table 3). Users reported that laboratory results were either partly or completely unavailable. In addition, some users reported that the records of certain health care visits or units did not seem to have been entered into the system. Some responses said that records of private health care providers, such as occupational health care, were missing. Users also reported that some information had disappeared, or they would like to see their health information from the years before the introduction of My Kanta.

Difficulty finding information in the service was frequently reported to be a problem in the main category *Difficulty navigating the service and searching for information* (Table 3). Some respondents did not specify what information they could not find, whereas others mentioned laboratory results or records of a particular health care visit. Some users thought that the service was badly arranged. For example, layout, menus, and headings were considered unclear, which complicated their ability to navigate within the service. Some users reported that switching between different pages did not work properly, as they could not move back to the previous page but instead were transported to the starting page.

Responses in the main category *Health data viewable with a delay* refer to the feeling that there are delays in health data being downloaded into the service (Table 3). Some respondents did not specify what health data they meant, whereas others mentioned records of health care visits or laboratory results.

### Potential Improvements

Of My Kanta users, 39.9% (327/820) reported at least one potential improvement in the service (Table 4). The most common suggestions for improvements were that the health data provided in the portal should be more comprehensive (89/327, 27.2%) and navigating the service and searching for information should be made easier (71/327, 21.7%). Some users suggested new functions that could be incorporated into the service (51/327, 15.6%) or would like health data to be entered more promptly into the service (47/327, 14.4%).

**Table 4:** Potential improvements proposed by users.

Proposed improvements^a^		Respondents (n=327), n (%)
**More comprehensive health data**		89 (27.2)
	Data from all health care units should be available	—^b^
	All data viewable in the service	—
	Records of health care visits in more detail	—
	Old data should be incorporated into the service	—
**Facilitating navigation in the service and searching for information**		71 (21.7)
	Data grouping more clearly	—
	Clearer layout	—
	Simplification of the service	—
	Use guidance	—
**More functions incorporated into the service**		51 (15.6)
	Secure messaging	—
	Notifications	—
	Vaccinations	—
	More information about medicines	—
	Opportunity to correct erroneous information	—
**Faster downloading of health data**		47 (14.4)
	Laboratory results entered into the service with a shorter delay	—
	Record of health care visits into the service with a shorter delay	—
	Data entered into the service with a shorter delay	—
**Easier monitoring of prescriptions**		33 (10.1)
	Certain^c^ prescriptions should be removed	—
	Clearer order for prescriptions	—
	Clearer prescription information	—
**Health data in plain language**		23 (7)
	Diagnosis, terms, and medical reports in easy-to-comprehend language	—
	Laboratory results in easy-to-comprehend language	—
**Easier logging in and mobile use**		22 (6.7)
	Other ways^d^ to log-in than web banking codes	—
	Simplifying the log-in procedure	—
	Mobile app should be introduced	—
**Making it easier to act on behalf of others**		19 (5.8)
	Opportunity to act on behalf of dependents aged 10-18 years^e^	—
	Opportunity to act on behalf of adults (>18 years)^e^	—
**Other**		26 (8)
	Improvements in prescription renewals	—
	Improvements in printing out information	—
	Data protection	—

^a^From each main category, the most common subcategories are reported in the table.

^b^Not available.

^c^For example, invalid or noncurrent prescriptions.

^d^For example, username and password or PIN code.

^e^In Finland, the age of majority is 18 years.

Responses in the main category *More comprehensive health data* related to health data that is or should be in the portal (Table 4). These responses included the view that health data from all visits or units should be added to the service. Some respondents did not specify from which units’ data were missing, whereas others mentioned as an example of private health care providers or oral health care. Many users wanted the laboratory results to be viewable in the service. Some of these respondents had no laboratory results in the service, whereas for others, they were only partially available. Some users wanted records of health care visits to be more detailed. A few respondents wanted data from the years before My Kanta or data that had disappeared to be added to the service.

In the main category *Facilitating navigation in the service and searching for information*, some respondents wanted the data to be grouped more clearly (Table 4). It was also suggested that there could be clear sections for different data, such as laboratory results, data from certain units, or the latest data. Some respondents wanted data to be arranged in a clearer chronological order. Some thought that searching for particular information should be made easier and the layout of the service clearer. Specifically, the headings and menus were considered to be unclear. Some users wanted the service to be simpler. Others reported that they would like to have more guidance in using the service. A few would like to receive face-to-face guidance, whereas others thought that some kind of manual or more general information should be provided.

The main category *More functions incorporated into the service* included responses proposing the addition of new functions to the portal (Table 4). Some users wanted secure messaging between patients and health care providers. It was considered an advantage if patients could ask physicians or nurses about their care or medicines via the service, or, in the case of prescription renewals, if they could send messages to their physician. Respondents also proposed the introduction of novel notifications, for example, an SMS text message to a mobile phone informing them when prescriptions had to be renewed or when new records were downloaded to the portal. Some users wanted more information about medicines to be included in the portal, such as drug interactions or costs. It was also suggested that vaccinations should be included. Some users wanted to have an opportunity to correct erroneous information via the service or to report incorrect information.

In the main category *Faster downloading of health data*, some respondents wanted to see laboratory results, records of health care visits, or data in general to be viewable in the service more promptly (Table 4). A few respondents wanted data to be entered into the service without delay or with a shorter delay, so they could use the information at their next appointment.

## Discussion

### Principal Findings and Comparison With Prior Work

In this study, more pharmacy customers reported benefits than problems or potential improvements when using the nationwide patient portal My Kanta. Users consider My Kanta beneficial because health and prescription information can be easily viewed and monitored, and prescription renewals can be submitted via the service. In general, the fact that all these services are available regardless of time and place, for example, in the privacy of the patient’s home, is regarded as a benefit. However, the system is not perfect, and some drawbacks were reported. Ideas for improvement mainly concerned certain frequently mentioned problems. The results of this study are mostly in line with those of previous studies investigating users’ experiences regarding the benefits, problems, and potential improvements of patient portals [3-11]. This suggests that there are no major differences in users’ experiences between nationwide and organization-specific portals, although these portals differ slightly from each other in terms of content and functions.

The My Kanta functions rated most beneficial, that is, viewing health data and prescriptions and renewing prescriptions, are also the most widely used [16]. According to a study conducted in 2014 among the Finnish population on the use of electronic services in health care, respondents said that in the future, they would like to have access to their laboratory test results, health records, and prescriptions, and an opportunity to submit renewal requests [24]. Thus, as these options are now available, individuals naturally regard them as the most beneficial, and these functions are most extensively used. The present results also indicate that My Kanta met users’ expectations well. In addition, the portal has achieved its intended goal of making it easier for patients to obtain their health information and hence participate in their own care.

In line with previous studies [3-7,9-11], the ability to view one’s own health data was regarded as the most beneficial function of the patient portal. However, users reported some problems that may discourage the use of health data and thus patient engagement. Some users found the language used in their health records too difficult to understand. This is also a finding from previous studies conducted in Finland [16] and other countries [3,5,10,11]. My Kanta is a nationwide portal that can be used by anyone living in Finland with an ID for electronic services. It is important that the language used in the portal is plain enough to allow everyone to easily understand the recorded data. Physicians should be informed about this finding so that they can avoid writing reports with professional terminology that laypeople will not understand. It would also be useful if My Kanta contained explanations of laboratory test abbreviations and reference values.

To increase and facilitate patient engagement, there are other issues that require attention. Many users reported that not all their health data, especially laboratory results and data from certain health care units, were available. This prevents patients from gaining a comprehensive picture of their health and care. Health data from public health care started to be included in My Kanta in 2013 and from private health care in 2016 [12]. It may be that at the time of this study, not all units had started to record their data in My Kanta, as this took place step by step. It should also be noted that the portal is continuously developing and that the amount of health data that can be accessed is constantly increasing [26]. For example, since the questionnaires were returned, oral health care data and vaccinations have begun to be downloaded to the portal. Furthermore, there is a possibility that data on some health care visits or units are delayed as health care professionals want to discuss the significance of findings with the patient before the data are made available in My Kanta [27]. In Finland, there is no exact time period determining when certain data should be entered in My Kanta; instead, this is left to the discretion of health care units or professionals. In some cases, records can be delayed indefinitely if their availability is seen as a risk to the patient’s life or care. It would be important to inform patients about these delay procedures as many users wanted their health data to be downloaded to My Kanta more promptly. The wish of patients to have their health data downloaded to the portal without delay has also emerged in studies conducted in other countries [3,9,11]. In contrast, in a study conducted in the Netherlands, users did not want their health data to be downloaded to the portal before they had visited a physician [5]. Some users may not have the ability to understand information, such as the meaning of laboratory results, and thus, delaying the data may be necessary to prevent unnecessary concerns. Nevertheless, it may be that patients are unaware of the reasons for a delay, and they should be informed accordingly, for instance, when visiting the health care provider.

Users also stated that some data were difficult to find in My Kanta. This was also highlighted in a previous study that investigated My Kanta usability [16]. In this study, many suggestions were made to make searching for information easier, for example, organizing data in the portal in different ways. Users also wanted the service to be simpler, especially headings. However, many of the suggestions differed from each other, so the first step in making information easier to find could be better instructions on the use of the portal, which was also the wish of users. It is evident that further investigations should be conducted to determine the optimal information search strategy. At the moment, Kanta pages on the internet include a considerable amount of self-guidance. There is a My Kanta web-based course including guidance videos and instructions to acquaint users with My Kanta functions and how to start using the portal [28]. Clients can also request help from the customer service [29]. However, as the wishes for easier use of the portal and more guidance were mentioned in many responses and as some users reported they were unaware of the content of My Kanta, it can be assumed that this information and guidance have not reached all citizens. A wish for further guidance on My Kanta use was also reported in a previous study [16]. As suggested in this study, it may be necessary to provide face-to-face guidance or a manual.

Opportunities to view and renew prescriptions emerged as the most commonly experienced benefits, which is a finding at odds with previous studies [3,5,7,10]. This difference may be due to the fact that there are only few countries where the use of e-prescriptions is as widespread as in Finland, where all prescriptions have been issued electronically since 2017 [30]. In Finland, My Kanta is the only way for patients to view and monitor their prescriptions, other than by visiting or calling health care units or pharmacies. In addition, the survey respondents were pharmacy customers who used prescription medicines, which may have biased the result. Although users experienced the ability to view prescriptions as beneficial, some suggested ways to make prescription monitoring easier. Respondents wanted to see prescriptions not in use removed from the service or the opportunity to arrange prescriptions or hide unnecessary prescriptions. This would simplify the overall view of their prescriptions in the service. Kanta Services is developing a national medication list [31]. This will be an up-to-date list of currently used medicines to be included in My Kanta, which may help patients to obtain an overview of their medication. Users also had difficulty understanding prescription information, especially valid dates and amounts of outstanding medicines. Health care professionals should explain this information to patients in health care units and pharmacies.

In this study, prescription renewal via My Kanta was experienced as easy, and some users stated that they now visited or called health care providers less often because they were able to send renewal requests via the portal. This saves time for health care professionals, which is one of the aims of eHealth services [2]. However, the renewal of prescriptions without any communication between the patient and health care professionals may pose risks for pharmacotherapy monitoring. More studies are therefore needed on prescription renewal via My Kanta so that the overall impact of this function on health care professionals’ workload and pharmacotherapy monitoring can be evaluated.

Users also wanted to see new information or functions included in the portal. This is in line with previous studies [3,4,8,10,11], indicating that portal users want all their health-related data to be available via these services. It could be argued that users would prefer patient portals to be web-based places where they could deal with many aspects of their health and medication. Communicating with health care professionals and notifications were the most often desired new functions. In Finland, some private health care providers have their own organization-based patient portals, which include communication features. It is common worldwide for these functions to be part of organization- or area-specific portals, and when available, these are also some of the most beneficial functions reported by portal users [3-7,11]. However, they have not been implemented in nationwide portals [9,10]. A communication function could be a useful way to enhance patient engagement and decrease the need for contact with health care. However, this might increase the workload of health care professionals, and therefore, before such a function is implemented in a nationwide patient portal, its impacts would need to be scrutinized in detail. The possibility of including notifications in My Kanta, as suggested in this survey, should also be evaluated. Users also wanted an opportunity to correct erroneous information on the portal. Some users reported that they had noted that their own data were incorrect or that there was some other person’s data in their portal. This problem was also mentioned in a previous study on My Kanta use [16]. In Finland, the procedure for correcting erroneous information is for the patient to contact the health care unit where the incorrect information has been entered and ask them to correct the error [13]. Evidently, this procedure is regarded as inconvenient.

### Strengths and Limitations

This study has both strengths and limitations. The findings are based on a nationwide patient portal that has been widely adopted by the Finnish population. By handing out the questionnaires from the pharmacies after dispensing the prescription medicine, we reached our target group (ie, medicine users who potentially have a need to use My Kanta). People who have prescription medicines also have contacts in health care and hence health data recorded in the service. Thus, medicine users represent a population with both health data and prescriptions recorded in My Kanta. This makes it a good population to study the use of My Kanta service. However, this target group may bias the results concerning prescriptions, as all respondents used prescription medicines.

The study sample was large and included pharmacy customers from all parts of the country. Evaluating the representativeness of the results is challenging because there are no comparable statistics on the characteristics of Finnish pharmacy customers. As the customers were recruited anonymously, we have no knowledge of the characteristics of those who declined to participate or those who did not return the questionnaires. The response rate in this study (994/2866, 34.68%) was low. However, the respondents’ characteristics (age, gender, education, and region) were fairly similar to those in earlier Finnish studies of pharmacy customers conducted using the same method with better response rates (40%-44%) [18,32].

The questions reported in this study were not validated measures. However, the questions were based on previous studies, with minor revisions [25,33]. In addition, both face validity and pilot tests were conducted. Many users (311/820, 37.9%-667/820, 81.3%) responded to the open-ended questions presented in this study. The reliability of categorization was ensured by the fact that 2 researchers conducted an inductive content analysis. Contradictory categorizations were discussed in the research group to obtain a consensus.

### Conclusions

Finnish pharmacy customers experience more benefits than problems or potential improvements when using the nationwide patient portal My Kanta. They perceive the portal as an easy and beneficial way to view their health data and prescriptions and to renew their prescriptions. However, portal users wanted the service to include more information and functions, and for data searches to be made easier. Fulfilling these wishes could encourage even greater use of the portal and help patients use the data recorded there. These improvements could allow patients to become more involved in their own health and care.

## Appendix

Multimedia Appendix 1 Questionnaire survey for pharmacy customers regarding the My Kanta service.
